# Treatment outcomes of carotid artery stenting with two types of distal protection filter device

**DOI:** 10.1186/2193-1801-3-132

**Published:** 2014-03-08

**Authors:** Minoru Iko, Hiroshi Aikawa, Yoshinori Go, Kanji Nakai, Masanori Tsutsumi, Iwae Yu, Taichiro Mizokami, Kimiya Sakamoto, Ritsuro Inoue, Takafumi Mitsutake, Ayumu Eto, Hayatsura Hanada, Kiyoshi Kazekawa

**Affiliations:** Department of Neurosurgery, Fukuoka University Chikushi Hospital, 1-1-1 Zokumyoin, Chikushino, Fukuoka, 818-8502 Japan

**Keywords:** Carotid artery stenting, Cerebral infarction, Diffusion-weighted magnetic resonance imaging, Distal protection filter device

## Abstract

**Purpose:**

Preventing cerebral embolism from debris produced during carotid artery stenting (CAS) is important. This study compared the treatment outcomes of CAS using two types of filter-based embolic protection devices currently in use in Japan.

**Materials and methods:**

We assessed 121 consecutive cases of CAS performed with FilterWire EZ™ between July 2010 and November 2012 and 37 consecutive cases of CAS performed with the Spider FX™ between November 2012 and June 2013. A Carotid Wallstent™ was used in all cases. The incidence of positive lesions on diffusion-weighted magnetic resonance imaging (DWI) and stroke were compared between the groups.

**Results:**

Postoperative DWI-positive lesions were observed in 38 (31.4%) and 14 (37.8%) patients in the FilterWire and Spider groups, respectively. In the FilterWire group, complications were transient ischemic attacks in 3 (2.5%) patients, cerebral infarction in 2 (1.7%) patients (1 patient each with minor and major stroke), and cerebral hemorrhage due to hyperperfusion syndrome in 1 (0.8%) patient. In the Spider group, except for cerebral infarction (minor stroke) in 1 (2.7%) patient, no complications were observed. No significant differences were observed in the incidence of complications between the groups.

**Conclusion:**

FilterWire EZ and Spider FX are comparable in terms of treatment outcome.

## Background

Carotid artery stenting (CAS), as a substitute for carotid endarterectomy (CEA), is rapidly becoming a popular treatment for carotid artery stenosis and has demonstrated a treatment outcome comparable to that of CEA (SPACE Collaborative Group et al. 2006). CAS is also more effective than CEA in patients at high risk of CEA complications (Yadav et al. 2004). Moreover, since its efficacy in medium-risk cases was recently reported (Brott et al. 2010), CAS is becoming a widely used alternative therapy to CEA. However, CAS is associated with the risk of intraoperative embolism. Preventing peripheral embolism is therefore important for improving the outcomes of this procedure.

Various methods have been proposed for embolism prevention and can be roughly divided into proximal protection, in which the proximal part of the lesion is blocked with a balloon, and distal protection, in which the distal part of the lesion is blocked with a balloon or fitted with a filter. Distal protection techniques are technically more convenient than proximal techniques, and a number of randomized trials have demonstrated the usefulness of embolic protection devices (EPDs) in preventing peripheral embolism.

In this article, we compare the outcomes of two types of EPDs, in particular, distal protection filter devices: FilterWire EZ™ (Boston Scientific, Natick, MA; hereafter referred to as FilterWire) and Spider FX™ (Covidien, Mansfield, MA; hereafter referred to as Spider).

## Methods

Informed consent was obtained from all patients after the nature of the procedures had been fully explained. Approval was obtained from the Fukuoka University Chikushi Hospital institutional review board. The Declaration of Helsinki was followed.

This study involved 158 consecutive cases of CAS performed at our hospital or affiliated institutions using either the FilterWire or Spider device. Cases performed by proximal protection or unprotected procedures were excluded. A total of 121 consecutive cases of CAS were performed with the FilterWire between July 2010 and November 2012 and the remaining 37 consecutive cases were performed with the Spider between November 2012 and June 2013. The Carotid Wallstent™ Monorail stent (Boston Scientific, Natick, MA) was used in all cases. The incidence of intraoperative flow impairment, ischemic cerebrovascular events, and hyperperfusion syndrome during the first 7 postoperative days and the incidence of positive lesions on diffusion-weighted magnetic resonance (MR) imaging (DWI) were compared between the two groups. Patients were considered eligible for CAS if they had a symptomatic stenosis ≥50% of the carotid artery or an asymptomatic stenosis ≥80% of the carotid artery, according to the criteria proposed in the North American Symptomatic Carotid Endarterectomy Trial (NASCET) (North American Symptomatic Carotid Endarterectomy Trial Collaborators 1991).

Before the procedure, all patients underwent MR plaque imaging with a Philips Ingenia 1.5 T scanner (Philips Healthcare, Heide, Netherlands). On black blood T1-weighted, black blood T2-weighted, and time-of-flight images, the signal intensity of the plaque within the carotid artery was compared with that of the ipsilateral sternocleidomastoid muscle. Plaques with intraplaque hemorrhage and a lipid-rich necrotic core were defined as vulnerable and those with fibrous tissue and calcification were defined as stable (Sakamoto et al. 2010).

All patients were treated with two of three oral antiplatelet drugs (aspirin 100 mg daily, clopidogrel 75 mg daily, or cilostazol 200 mg daily) for at least 5 days before the procedure. Under general anesthesia, an 8-Fr sheath was inserted into the femoral artery through which heparin (80 IU/kg) was infused intravenously to achieve an activated clotting time of ≥250 s. An 8-Fr guiding catheter was advanced into the common carotid artery on the affected side, and the FilterWire or Spider device was advanced through the narrowing portion into the distal part of the internal carotid artery. Angiography was then performed to confirm that antegrade blood flow was observed after the filter was unfolded. A stent was placed after balloon pre-dilatation, and post-dilatation was performed if residual stenosis ≥20% was observed, in accordance with the NASCET criteria. Angiography was also performed to identify any flow impairment. Flow impairment consisted of “stop flow”, defined as disrupted blood flow, and “low flow”, defined as delayed visualization of the internal carotid artery compared with the external carotid artery. If flow impairment was identified, 60 ml blood was aspirated from the vicinity of the protection device, followed by device removal. In the event of bradycardia or hypotension during the procedure, atropine sulfate or a vasopressor was used in the appropriate manner. After the operation, heparin neutralization was not performed and the puncture site was treated with a hemostatic device (Angio-Seal, St. Jude Medical. Inc., St. Paul, MN). The patient was then transferred to the intensive care unit or special care unit and, immediately after recovery from anesthesia, was monitored for any neurological symptoms.

All patients underwent head MR imaging upon the onset of new neurological symptoms or, for asymptomatic patients, at 1–3 days after operation to identify any new ischemic lesions in the brain. The Philips Ingenia 1.5 T MR imaging apparatus was used to obtain fluid-attenuated inversion recovery (FLAIR) and DWI images. DWI images were obtained with the following parameters: b value 1,000 s/mm^2^, repetition time/echo time/excitation 3803 ms/92 ms/1, 128 × 128 matrix, field of view 230 × 230 mm, 5 mm slice thickness, and 0.5 mm interslice gap, with a total acquisition time of 45 s. The parameters for FLAIR images were repetition time/echo time/excitation 11,000 ms/110 ms/1, 272 × 272 matrix, field of view 230 × 230 mm, 5 mm slice thickness, and 0.5 mm interslice gap, with a total acquisition time of 2 min 45 s. New cerebral ischemic lesions (DWI-positive lesions) were identified on FLAIR, DWI, or apparent diffusion coefficient maps, and the number of non-contiguous lesions was counted. Image analysis was performed by two neurosurgeons who did not perform the CAS procedures.

Ischemic neurological events were classified into transient ischemic attack (TIA; defined as neurological deficit symptoms that improved within 24 h), minor stroke (defined as prolonged neurological deficit symptoms persisting >24 h and a National Institute of Health Stroke Scale [NIHSS] score of ≤4), and major stroke (defined as prolonged neurological deficit symptoms persisting for >24 h and an NIHSS score of ≥5). Hyperperfusion syndrome was defined as the presence of postoperative intracranial hemorrhage with an increase in cerebral blood flow of >100% compared with the preoperative value, as determined by a cerebral blood flow test.

For statistical comparison of clinical characteristics between the two groups, the independent *t*-test was used for age and the degree of stenosis, while Pearson’s Chi-squared test was used for sex-related differences and the frequency of pre- and postoperative risk factors. The incidence of flow impairment, ischemic neurological events, and hyperperfusion syndrome was analyzed using Fisher’s exact test and that of postoperative DWI-positive lesions was analyzed using Pearson’s Chi-squared test. Significance was set at p ≤ 0.05.

## Results

The clinical characteristics of patients in both groups are summarized in Table 1. No significant differences were found between the groups with regard to patient demographics, and stent placement was successful in all patients in both groups. Direct puncture of the common carotid artery was performed in 2 patients in the FilterWire group and in no patients in the Spider group.

**Table 1:** **Baseline characteristics of patients who underwent carotid artery stenting with different filter-based embolic protection devices**

	FilterWire ^1^ (n = 121)	Spider ^2^ (n = 37)	p-value
Age, sex			
Mean age (years)	72.6 ± 9.0	71.1 ± 8.6	0.39*
Male	104 (84%)	29 (78%)	0.26**
Cardiovascular risk factor			
Diabetes mellitus	38 (31%)	13 (37%)	0.67**
Hypertension	96 (79%)	29 (78%)	0.89**
Dyslipidemia	58 (48%)	20 (54%)	0.51**
Atrial fibrillation	15 (11%)	4 (11%)	0.79**
History of cardiovascular disease	22 (18%)	6 (16%)	0.78**
Presenting symptom			
Cerebral infarction	53 (43%)	18 (48%)	0.60**
TIA^3^ or amaurosis fugax	11 (9.1%)	4 (11%)	0.75**
Asymptomatic	57 (47%)	15 (41%)	0.48**
Degree of carotid artery stenosis	77.5 ± 13.1	75.2 ± 14.6	0.39*

^1^FilterWire EZ™.

^2^Spider FX™.

^3^TIA, transient ischemic attacks.

*Independent *t*-test; **Pearson’s Chi-squared test.

Flow impairment was observed in 14 (11.6%) patients in the FilterWire group and in 1 (2.7%) patient in the Spider group. The incidence of flow impairment was lower, but not significantly, in the Spider group (p = 0.19).

Ischemic neurological events were observed in 6 (3.8%) patients in the entire study population. Of the 5 (4.1%) cases that occurred in the FilterWire group, 3 (2.5%) were TIA, 1 (0.8%) was minor stroke, and 1 (0.8%) was major stroke. The 1 (2.7%) case in the Spider group was minor stroke. Hyperperfusion syndrome was observed in 3 (2.5%) patients in the FilterWire group, of which 2 had only headache and the remaining 1 had extensive intracranial hemorrhage and required craniotomy. Hyperperfusion syndrome was also observed in 2 (5.4%) patients in the Spider group. Both patients had only headache and no neurological deficit symptoms were observed. Furthermore, no significant intergroup differences were observed in the incidence of ischemic neurological events (p = 1.00) or hyperperfusion syndrome (p = 0.33) (Table 2).

**Table 2:** **Flow impairment and postoperative stroke events in relation to the filter-based embolic protection device used**

	FilterWire ^1^ (n = 121)	Spider ^2^ (n = 37)	p-value
Flow impairment	14 (11.6%)	1 (2.7%)	0.19*
Postoperative stroke event			
TIA or amaurosis fugax	3 (2.5%)	0 (0%)	1*
Minor stroke	1 (0.8%)	1 (2.7%)	0.41*
Major stroke	1 (0.8%)	0 (0%)	0.49*
Total ischemic neurological events	5 (4.1%)	1 (2.7%)	1*
Hyperperfusion syndrome	3 (2.5%)	2 (5.4%)	0.33*

^1^FilterWire EZ™.

^2^Spider FX™.

*Fisher’s exact test.

Postoperative DWI-positive lesions were observed in 38 (31.4%) patients in the FilterWire group and in 14 (37.8%) patients in the Spider group, with no significant differences (p = 0.47). The number of patients who had 1, 2, and ≥3 DWI-positive lesions was 16 (13.2%), 9 (7.4%), and 13 (10.7%) in the FilterWire group and 5 (13.5%), 3 (8.1%), and 6 (12.3%) in the Spider group, respectively, with no significant differences between the groups (p = 0.96, p = 0.89, and p = 0.37, respectively) (Table 3). Of the 14 patients with flow impairment in the FilterWire group, 2 (14.2%) had ischemic neurological events and 8 (57.1%) had postoperative DWI-positive lesions. The incidence of these complications was higher than that in the 107 patients without flow impairment (ischemic neurological events in 3 (2.8%) and postoperative DWI-positive lesions in 30 (28.0%) patients), with a significantly higher incidence of postoperative DWI-positive lesions in patients with flow impairment (p = 0.10 and p = 0.03, respectively) (Table 4). Neither postoperative ischemic neurological events nor DWI-positive lesions were observed in those with flow impairment in the Spider group.

**Table 3:** **Magnetic resonance imaging after the procedure**

	FilterWire ^1^ (n = 121)	Spider ^2^ (n = 37)	p-value
New white lesions on DWI^3^	38 (31.4%)	14 (37.8%)	0.47*
No. of new white lesions			
1	16 (13.2%)	5 (13.5%)	0.96*
2	9 (7.4%)	3 (8.1%)	0.89*
≥3	13 (10.7%)	6 (12.3%)	0.37*

^1^FilterWire EZ™.

^2^Spider FX™.

^3^DWI, diffusion-weighted magnetic resonance imaging.

*Pearson’s Chi-squared test.

**Table 4:** **Flow impairment with the FilterWire EZ** **™**

	Flow impairment (n = 14)	Normal flow (n = 107)	p-value
Ischemic neurological events	2 (14.2%)	3 (2.8%)	0.10*
New white lesions on DWI^1^	8 (57.1%)	30 (28.0%)	0.03**

^1^DWI, diffusion-weighted magnetic resonance imaging.

*Fisher’s exact test; **Pearson’s Chi-squared test.

Of the 66 patients with unstable plaque in the FilterWire group, 8 (12.1%) had flow impairment, 3 (4.5%) had ischemic neurological events, and 27 (40.9%) had postoperative DWI-positive lesions. In comparison, of the 55 patients without unstable plaque in the FilterWire group, 6 (10.9%) had flow impairment, 2 (3.6%) had ischemic neurological events, and 11 (20.0%) had postoperative DWI-positive lesions, respectively, with a significantly higher incidence of postoperative DWI-positive lesions in patients with unstable plaque (p = 1.00, p = 1.00, and p = 0.02, respectively). Of the 23 patients with unstable plaque in the Spider group, 1 (4.3%) had flow impairment, 1 (4.3%) had ischemic neurological events, and 10 (43.5%) had postoperative DWI-positive lesions. Of the 14 patients without unstable plaque in the Spider group, no patients had flow impairment or ischemic neurological events and 4 (28.6%) had postoperative DWI-positive lesions. No significant intergroup differences were observed in the incidence of ischemic neurological events (p = 1.00) or postoperative DWI-positive lesions (p = 0.36) (Table 5).

**Table 5:** **Outcome for patients with vulnerable or stable plaques according to filter-based embolic protection device used**

FilterWire ^1^	Vulnerable plaque (n = 66)	Stable plaque (n = 55)	p-value
Flow impairment	8 (12.1%)	6 (10.9%)	1*
Ischemic neurological events	3 (4.5%)	2 (3.6%)	1*
New white lesions on DWI	27 (40.9%)	11 (20.0%)	0.02**
**Spider** ^**2**^	**Vulnerable plaque (n = 23)**	**Stable plaque (n = 14)**	**p-value**
Flow impairment	1 (4.3%)	0 (0%)	1*
Ischemic neurological events	1 (4.3%)	0 (0%)	1*
New white lesions on DWI^3^	10 (43.5%)	4 (28.6%)	0.36**

^1^FilterWire EZ™.

^2^Spider FX™.

^3^DWI, diffusion-weighted magnetic resonance imaging.

*Fisher’s exact test; **Pearson’s Chi-squared test.

## Discussion

The use of EPDs has been shown to be effective in CAS procedures (Kastrup et al. 2003, 2006) and these devices are therefore routinely used in CAS. EPDs are roughly divided into distal protection balloon (DPB) devices, which block blood flow in a peripheral artery with a balloon to prevent peripheral embolism caused by debris, and distal protection filter (DPF) devices, which capture debris with a filter placed in a peripheral artery. Kim et al. ( 2007) compared the outcome of CAS using the PercuSurge GuardWire™ DPB device (Medtronic, Minneapolis, MN) in 33 patients with that of CAS using the FilterWireEX/EZ DPF device with an Emboshield filter (Abbott Vascular, Santa Clara, CA) in 38 patients and reported a similar incidence of new ischemic lesions on DWI with both devices. Zahn et al. ( 2005) also compared the outcomes of CAS with DPB and DPF devices and found no significant differences in the incidence of TIA, minor stroke, or major stroke. Nevertheless, the fact that a decreased incidence of ischemic lesions has been reported with the FilterWire (Iko et al. 2013) suggests the superiority of DPF devices as EPDs.

In addition to the FilterWire and Spider, currently available DPF devices include the Angioguard™ XP (Cordis Corp, Bridgewater, NJ), RX Accunet™ (Abbott Vascular), Emboshield™ NAV6, and FiberNet™ (Lumen Biomedical, Inc., Plymouth, MN). Loghmanpour et al. ( 2013) compared the outcomes of these six DPF devices and reported that the overall incidence of TIA and stroke was 2.6% and 5.2%, respectively, and that although the RX Accunet accounted for about 60% of all devices used, there were no significant differences in clinical outcome between the six devices. However, the incidence of combined adverse events they reported was 5.4% with the RX Accunet, 12.3% with the FilterWire, and a relatively higher 17.8% with the Spider. On the other hand, Iyer et al. ( 2007) reported the incidence of combined adverse events with the RX Accunet, FilterWire, and Spider to be as low as 5.9%, 2.2%, and 2.1%, respectively, which is comparable to that in the present study. Different DPF devices have different properties in terms of capture efficiency, porosity, vascular resistance, wall apposition, pore density, and concentricity (Müller-Hülsbeck et al. 2002; Siewiorek et al. 2009; Loghmanpour et al. 2013). In particular, the Spider has been shown to have lower capture efficiency than other devices (Siewiorek et al. 2009). Meanwhile, Loghmanpour et al. also reported that clinical outcome is affected by the device’s porosity, wall apposition, and pore density but not by capture efficacy (Loghmanpour et al. 2013). This may explain why no significant differences have been found in treatment outcomes between the FilterWire and Spider. The Spider has a larger pore size than the FilterWire (110 μm vs. 70–200 μm) and, according to Siewiorek et al. ( 2009), is unable to capture small particles. In the present study, CAS with the Spider was associated with a higher but non-significant incidence of postoperative DWI-positive lesions than CAS with the FilterWire, which may be attributed to the different filter properties. Since the clinical outcome in the Spider group was similar to that reported by Iyer et al. ( 2007), it is likely that the small DWI-positive lesions caused by small debris not captured by the Spider did not progress to cerebral infarction with the potential to cause permanent neurological deficit symptoms.

The incidence of flow impairment in CAS with DPF devices is 6–30%, whereas that with the FilterWire or Spider is as low as 6–8% (Kwon et al. 2006; Roffi et al. 2008). In the present study, flow impairment was observed in 14 (11.6%) patients in the FilterWire group and in 1 (2.7%) patient in the Spider group, which is similar to the results of previous studies. Furthermore, normal blood flow in this study was re-established with no signs of subsequent vasospasm in any cases of flow impairment, indicating that the flow impairment was caused by the filter clogging and not by vasospasm. Although no significant differences were observed in the incidence of flow impairment between the groups, those patients with flow impairment in the FilterWire group were more likely to have ischemic neurological events and postoperative DWI-positive lesions, with a significantly higher incidence of postoperative DWI-positive lesions in patients with flow impairment than in those without. Casserly et al. ( 2005) reported a significantly higher incidence of stroke within 30 days of the operation in patients with flow impairment (9.3%), compared with that in patients with normal flow (1.7%), which is similar to the present results. Flow impairment occurs with clogging of the filter by debris produced during the CAS procedure and subsequent blockage of peripheral blood flow by the debris-filled filter. Thus, filters with higher capture efficiency appear to be more likely to cause flow impairment. The FilterWire has a smaller pore size and higher capture efficiency than the Spider (Siewiorek et al. 2009). Flow impairment causes debris to flow towards the distal part of the internal carotid artery, which may explain the increased incidence of ischemic neurological events and postoperative DWI-positive lesions in patients with flow impairment. The incidence of flow impairment with the Spider in the present study was low (2.7%), which is also likely to be related to the filter’s pore size and capture efficiency. These results might be explained by the fact that the device is poor at capturing small debris and thus is more likely to cause postoperative DWI-positive lesions and less likely to cause flow impairment and ischemic neurological events.

Unstable plaque contains apoptotic smooth muscle cells and infiltrating inflammatory cells, such as macrophages and lymphocytes, and is covered by a thinned and weakened fibrous capsule (Virmani et al. 2006). CAS for internal carotid artery stenosis with unstable plaque can cause plaque rupture and thrombus formation and thereby increase the risk of cerebral infarction. van Lammeren et al. ( 2011) have suggested that the increased aging of the population will lead to more CAS procedures being performed in patients with unstable plaque, thereby increasing the incidence of postoperative cerebral infarction. In the present study, the incidence of ischemic neurological events and postoperative DWI-positive lesions was higher in patients with unstable plaque in both groups, with significant differences in the FilterWire group. Sakamoto et al. ( 2010) compared the incidence of flow impairment and DWI-positive lesions after CAS in patients with and without unstable plaque and found a significant difference in the incidence of flow impairment but not in that of DWI-positive lesions. Although the results of their study cannot simply be compared with ours because they used a different type of DPF device (Angioguard XP), it is likely that unstable plaque is closely associated with the development of flow impairment and postoperative DWI-positive lesions. It therefore seems reasonable to consider the use of proximal protection or carotid endarterectomy in patients with unstable plaque and who are thus at high risk of CAS complications.

The Carotid Wallstent Monorail stent (diameter, 10 mm; length, 24 mm) was used in all cases in this study. Although stenting was successful in all cases, the use of an open-cell stent is preferable in patients with high lesion tortuosity in the carotid artery (Myouchin et al. 2013). In addition, the use of different type and size stents is recommended depending on the length and shape of the lesion in individual patients (Bates et al. 2007). However, in accordance with our CAS policy, our hospital and affiliated institutions use the same technique and device as often as possible to enable us to compare the outcomes of CAS. Although we have used open-cell stents in some patients, placement of the Carotid Wallstent Monorail stent has, in our experience, been successful, even in patients with high lesion tortuosity in the carotid artery and slight displacement of the flow path when applying pressure from the surface of the cervix and repositioning the guiding catheter in the peripheral direction. In addition, we also select the Carotid Wallstent Monorail stent for its ability to prevent plaque protrusion.

We initially used the FilterWire as a distal protection filter device for CAS but experienced difficulty in advancing it through severely calcified or curved lesions. Since the Spider could be advanced more easily through lesions and showed a comparable clinical outcome, this prompted us to use the Spider in CAS. However, in addition to DPF devices such as the two we used, several other options are available as EPDs for CAS, including DPB devices (e.g., the PercuSurge GuardWire) and the proximal protection method. We hope that the findings of the present study will provide useful information for determining the treatment strategy for carotid artery stenosis.

This study has some limitations. It was not a randomized study: the first series of patients were treated with the FilterWire and the subsequent series with the Spider following favorable initial outcomes obtained with the latter device. Although the possibility that improved skills in the CAS procedure contributed to the improved outcome was not taken into account, given that both the FilterWire and Spider devices are filter-type EPDs, the impact of greater technical proficiency that occurs with a learning curve is considered small.

## Conclusions

The FilterWire was associated with a higher incidence of flow impairment and postoperative DWI-positive lesions than the Spider in patients with unstable plaque. However, as no significant difference in the incidence of ischemic neurological events was apparent between the FilterWire and Spider groups, the devices are considered to be comparable in terms of treatment outcome.

## Authors’ information

Minoru Iko is a member of The Japan Neurosurgical Society, The Japanese Society for Neuroendovascular Therapy, and The Japan Stroke Society. Kiyoshi Kazekawa is Chief of the Department of Neurosurgery, Fukuoka University Chikushi Hospital.

## Abbreviations

CAS: Carotid artery stenting
CEA: Carotid endarterectomy
DWI: Diffusion-weighted magnetic resonance imaging
EPDs: Embolic protection devices
MR: Magnetic resonance
NASCET: North American symptomatic carotid endarterectomy trial
FLAIR: Fluid-attenuated inversion recovery
TIA: Transient ischemic attack
NIHSS: National Institute of Health Stroke Scale
DPB: Distal protection balloon
DPF: Distal protection filter.
